# Association of frailty with cardiovascular and all-cause mortality in community-dwelling older adults: insights from the Chinese longitudinal healthy longevity survey

**DOI:** 10.3389/fcvm.2024.1499099

**Published:** 2024-12-23

**Authors:** Hui Gao, Qiaoli Ma, Jiahai Li, Qinghui Zhang

**Affiliations:** ^1^ Department of Cardiovascular Medicine, The First People’s Hospital of Shangqiu, Shangqiu, China; ^2^ Graduate School, Dalian Medical University, Dalian, China; ^3^ Department of Cardiovascular Medicine, Central Hospital of Zibo, Zibo, China; ^4^ Department of Cardiovascular Medicine, The First People’s Hospital of Qinzhou, Qinzhou, China; ^5^ Department of Hypertension, Henan Provincial People’s Hospital, Zhengzhou, China

**Keywords:** Chinese older adults, frailty, cardiovascular disease mortality, all-cause mortality, geriatric epidemiology, competing risk

## Abstract

**Background:**

Previous studies suggest that frailty increases the risk of mortality, but the risk of cardiovascular disease (CVD) and all-cause mortality in Chinese community-dwelling older adults remains understudied. Our aim was to explore the effect of frailty on cardiovascular and all-cause mortality in older adults based on a large-scale prospective survey of community-dwelling older adults in China.

**Methods:**

We utilized the 2014–2018 cohort of the Chinese Longitudinal Healthy Longevity Survey and constructed a frailty index (FI) to assess frailty status. Propensity score matching was used to equalize the baseline characteristics of participants to strengthen the reliability of the findings. Hazard ratios and 95% confidence intervals (CIs) were estimated using multivariate Cox models, adjusting for potential confounders, to assess the association between frailty and cardiovascular and all-cause mortality. The relationship between frailty and cardiovascular mortality was further explored using a competing risk model considering death as a competing event. The dose–response relationships between them were estimated using restricted cubic spline models.

**Results:**

The results of the multivariate Cox model found that the frailty group had a higher risk of CVD mortality (1.94, 95% CI: 1.43–2.63) and all-cause mortality (1.87, 95% CI: 1.63–2.14) in compared with the non-frailty group. The multivariate competing risks model suggested a higher risk of CVD mortality in the frailty group (1.94, 95% CI: 1.48–2.53). The analysis found no non-linear relationship between FI and the risk of CVD mortality but a non-linear dose–response relationship with the risk of all-cause mortality.

**Conclusions:**

Frail older adults demonstrated a stronger risk of CVD and all-cause mortality. Reversing frailty in older adults is therefore expected to reduce the risk of death in older adults.

## 1 Introduction

Globally, cardiovascular disease (CVD) remains one of the most common causes of death among older people (1, 2). This includes coronary heart disease, stroke, hypertension, and heart failure. Older people, as a high-risk group for impaired health, are similarly more susceptible to chronic diseases and a variety of other diseases, making all-cause mortality more common among older people. With the general trend of population aging, the incidence of CVD and mortality among older persons has increased accordingly (3, 4). Specifically, ischemic heart disease was the second leading cause of death among the Chinese population in 2016. In addition, China reported higher CVD age-standardized mortality and disability-adjusted life years (DALYs) than the United States and Japan in 2019 (5, 6). This poses a challenge to the management of cardiovascular health among older persons.

Frailty is a complex physiological state usually characterized by a reduced ability to tolerate stressors, resulting in weakened overall functioning and increased vulnerability. It is commonly observed in older adults (7–9). Biological age, estimated based on an individual's physiologic and health status, serves as an important biological indicator for assessing individual aging (10, 11). It is also thought to be associated with higher morbidity and mortality rates and reduced life expectancy (12). The frailty index (FI) is widely used to evaluate biological age, which assesses the frailty status as a continuous score by summing signs, symptoms, disabilities, and diseases (8, 13). Previous studies have shown that FI performs better in predicting mortality and, more precisely, defines the risk of developing adverse outcomes (14–16).

A significant amount of research has explored the relationship between frailty and mortality risk. Most meta-analyses and systematic reviews that consider all-cause mortality as the primary endpoint have concluded that frailty increases the risk of death (15, 17). Recently, more attention has been paid to frailty-induced cause-specific mortality, such as cardiovascular mortality, but most of them have focused on non-Chinese regions (18–20) or only on older adults with underlying diseases such as diabetes (21–23). There is still limited research on community-dwelling and older adults in China. The study by Fan et al. on frailty and idiosyncratic mortality in China also included only adults aged 30–79 years and did not include the more elderly population (24).

Our study aimed to explore the association between frailty and CVD and all-cause mortality in older adults using data from a highly representative national follow-up survey of older adults in China. Furthermore, we explored whether there is a potential dose–response relationship between the FI, which evaluates frailty status, and the risk of CVD and all-cause mortality. We hypothesized an association between frailty and mortality in older adults. Proposing effective interventions may help reduce the risk of CVD incidence and mortality in older adults.

## 2 Methods

### 2.1 Study design and participants

**Figure 1:**
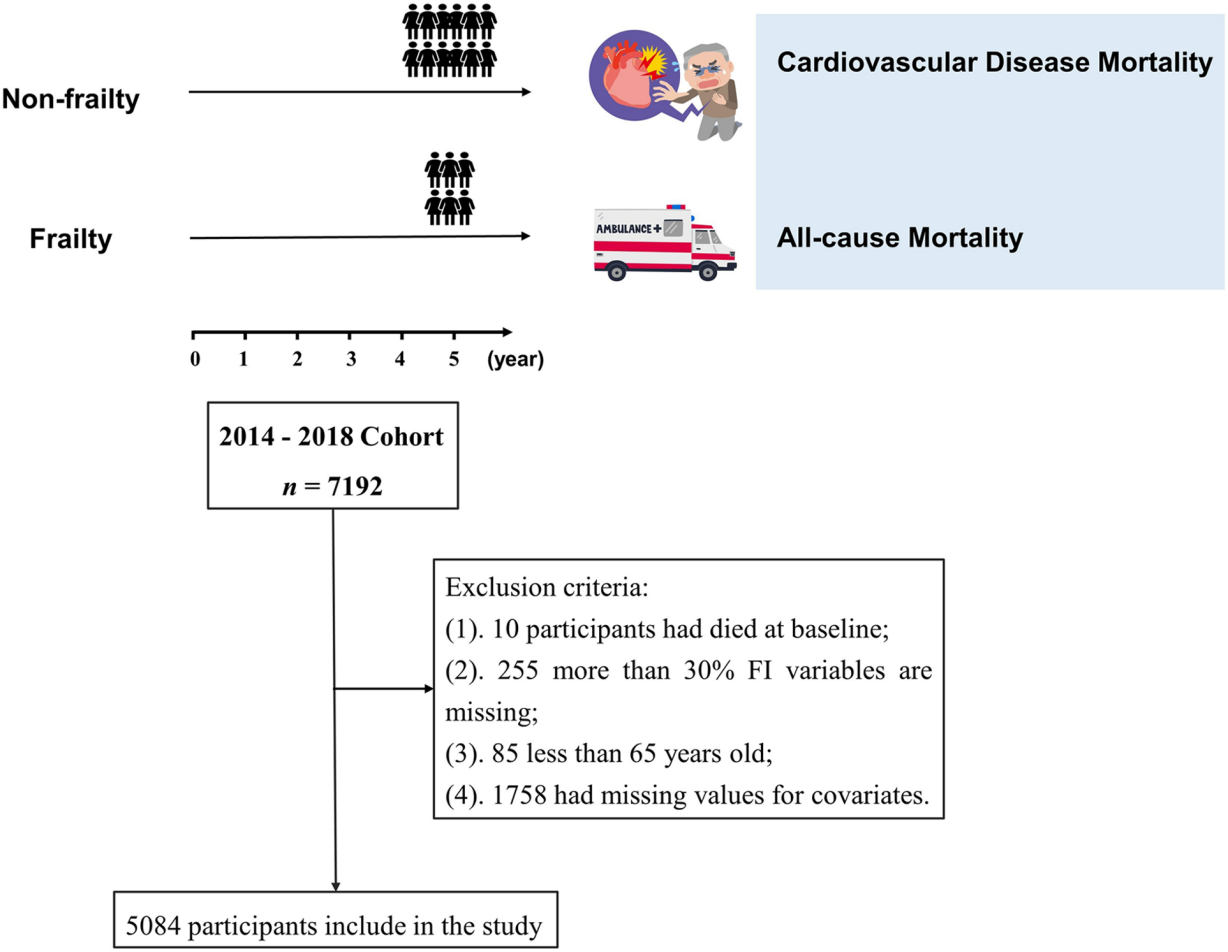
A flowchart for this study.

The data for this study were obtained from the Chinese Longitudinal Healthy Longevity Survey (CLHLS). Detailed information about this survey has been described elsewhere (25). As the database only investigated the cause of death of participants in 2018, the 2014–2018 cohort was selected for this study, with a total of 7,192 participants. The cohort dataset was cleaned according to the following exclusion criteria: (1) 10 participants who had died at baseline, (2) 255 participants with missing frailty indicators or more than 30% missing FI variables, and (3) 85 participants who were younger than 65 years. In addition, we excluded 1,758 participants with missing values for covariates from the main analyses. Ultimately, 5,084 older adults were included in the study. A detailed data-cleaning flowchart for this study is shown in Figure 1. All participants or their proxies signed a written informed consent form. The study was approved by the Biomedical Ethics Committee of Peking University (IRB00001052-13074). All methods were performed in accordance with the Declaration of Helsinki and relevant guidelines.

### 2.2 Outcome measures

The primary outcome indicator of our study was CVD mortality, referring to deaths where the cause was registered as circulatory. The CLHLS project interviewed relatives of older adults and registered the specific cause of death (including circulatory diseases, tumors, etc.) and the exact time of death interviewed in the 2018 survey of older adults who died. The secondary outcome was all-cause mortality, including all causes of death. Follow-up time was calculated as the number of days from the date of the baseline survey interview to the date of death registration or administrative review. Deaths were recorded by professionals according to the 10th edition of the International Classification of Diseases (ICD-10).

### 2.3 Exposure of interest

The exposure indicator of interest in this study was the frail status of older adults, which was assessed by the FI. The FI was constructed according to a standardized procedure and included 39 variables (26). Each variable was defined as binary or ordinal, with assigned values ranging from 0 to 1 (Supplementary Table S1). The FI was computed by dividing an individual's weighted count of actual cumulative deficits by the total number of healthy defects (27). We excluded individuals for whom data were missing for all defect indicators and those for whom data were missing for more than 30% of the defects. We defined FI ≥ 0.25 as frailty and FI < 0.25 as non-frailty (28).

### 2.4 Evaluation of covariates

Combining the specificity of the CLHLS and the findings of previous studies, three main components of potential confounders were considered in our study: socio-demographic factors, socio-behavioral factors, and dietary habits. The acquisition of all covariates was performed using face-to-face interviews and measurements. Socio-demographics used as covariates included age (categorized into three subcategories: 65–79, 80–89, and ≥90 years), sex, ethnicity, residence (categorized into city, town, and rural), co-residence (including family members, alone, and in nursing homes), education level (uneducated, primary education; junior and above level), total household income in the last year (>20,000 for high income, ≤20,000 yuan for low income), marital status (as a binary variable: married and other, including unmarried, divorced, and widowed), and the body mass index (BMI) at baseline (18.5–23.9 kg/m^2^ for normal individuals, <18.5 kg/m^2^ for underweight individuals, 24.0–27.9 kg/m^2^ for overweight persons, and ≥28.0 kg/m^2^ for obese). Socio-behavioral factors included smoking (never, former, and present), drinking status (never, former, and present), currently exercising regularly (question them, “exercise or not at present?”), regularly doing physical labor (question them, “have you done physical labor regularly?”), participating in social activities (question them, “do you take part in some social activities at present?”), and having a pension. Dietary habits mainly included fruit and vegetable intake (categorized as every day, often, occasionally, and never), meat intake (categorized as every day, per week, occasionally, and never), and the type of edible oil (including animal oils and vegetable oils).

### 2.5 Statistical analysis

To balance the differences in baseline characteristics between the two exposure groups (non-frailty and frailty), we used a 1:1 nearest-neighbor matching method based on the propensity score (PS) approach to achieve this using a caliper value = 0.1. This method removes confounding bias from observational cohorts that do not receive the benefit of randomization; it involves pairing each individual in the observation group with the closest matching individual from the control group until a match is found for each individual in the observation group (29, 30). PS was calculated using the logistic regression model for each participant and was used to match patients with similar confounder distributions (30). These were implemented using the R package “MatchIt” (31). Between-group balance was assessed for all covariates using the standardized mean difference (SMD), which compares the difference in means in terms of the combined standard deviation (32). A smaller SMD indicated less variation, and an SMD < 0.1 was considered better for balance (32). Furthermore, examining the distribution of PS in the original and matched groups, as well as the PS overlap, contributes to a quick and intuitive diagnosis of balance.

Differences in baseline characteristics between the two exposure groups were compared in the unmatched and matched datasets, respectively. All covariates were reported as categorical variables, presented as numbers (%), with comparisons made using chi-square tests. The incidence rates per 1,000 person-years and their 95% confidence intervals (CIs) were calculated. To estimate the association between frailty and CVD mortality and all-cause mortality, we used univariate and multivariate Cox proportional risk models to calculate hazard ratios (HRs) and 95% CIs. Moreover, considering CVD mortality as the major event and non-CVD death as a competing event, we used univariate and multivariate competing risk models to explore the association between frailty and CVD mortality. Model 1 is unadjusted, model 2 is adjusted for socio-demographic characteristics, model 3 is additionally adjusted for socio-behavioral factors, and model 4 is additionally adjusted for dietary habits. Proportional hazards (PH) assumption was checked for each model (including competing risk models) using the Schoenfeld residuals method. Each model satisfied the assumption of equal proportional risk (*P* > 0.10), and no collinearity was found between any of the variables [variance inflation factor (VIF) < 10]. The number of knots in the restricted cubic spline (RCS) model was selected based on the principle of maximizing the *R*^2^ of the model. The four-knots RCS regression model with FI = 0.1 as the reference level was used to explore the non-linear relationship between FI and CVD and all-cause mortality. In addition, we conducted subgroup and interaction analyses according to age, BMI, and sex.

Sensitivity analysis is likewise necessary. We performed the following analyses separately: (1) the frailty was divided into a three-categorical variable [robust (FI < 0.1), pre-frailty (0.1 ≤ FI < 0.25), and frailty (FI ≥ 0.25)] based on the majority of experience from previous studies; (2) we performed association analyses using inverse probability treatment weighting (IPTW) based on propensity scores; and (3) we used the multiple imputation method to generate 10 completely filled datasets, followed by estimation of HR and 95% CI according to Rubin's rule.

All statistical analyses were performed using R language software (version 4.2.2). Differences were considered statistically significant at *P* < 0.05.

## 3 Results

### 3.1 Characteristics of participants

Overall, 5,084 older adults were enrolled in the study, with 78.1% in the non-frailty group and 21.9% in the frailty group. A total of 1,482 deaths were recorded, of which 280 were due to CVD. The average age of these older adults was 85 (standard error: 10.1) years. The median follow-up times for CVD mortality and all-cause mortality were 4.03 (95% CI: 4.02–4.05) and 4.13 (95% CI: 1.13–1.14) years (17,597.7 person-years), respectively (Supplementary Figure S1). The incidence rates per 1,000 person-years for the cohort were 15.91 (95% CI: 12.47–19.35) and 84.22 (95% CI: 76.58–91.85), respectively. The baseline characteristics of the participants are shown in Supplementary Table S2. In the frailty group, there were higher proportions of advanced age (≥90 years), obesity, female gender, nursing home residence, lack of education, infrequent physical activity, absence of physical labor, no social activity, and small intake of fruits, vegetables, and meats compared with the non-frailty group (all *P*-values <0.05). We balanced differences in covariates between the two groups using propensity score matching (PSM). The individual PS distributions in the matched datasets showed large overlapping (Supplementary Figure S2). All covariates were well balanced, with SMD < 0.1, indicating no statistically significant difference between groups (Supplementary Figure S3).

### 3.2 Association between frailty and cardiovascular disease and all-cause mortality

**Figure 2:**
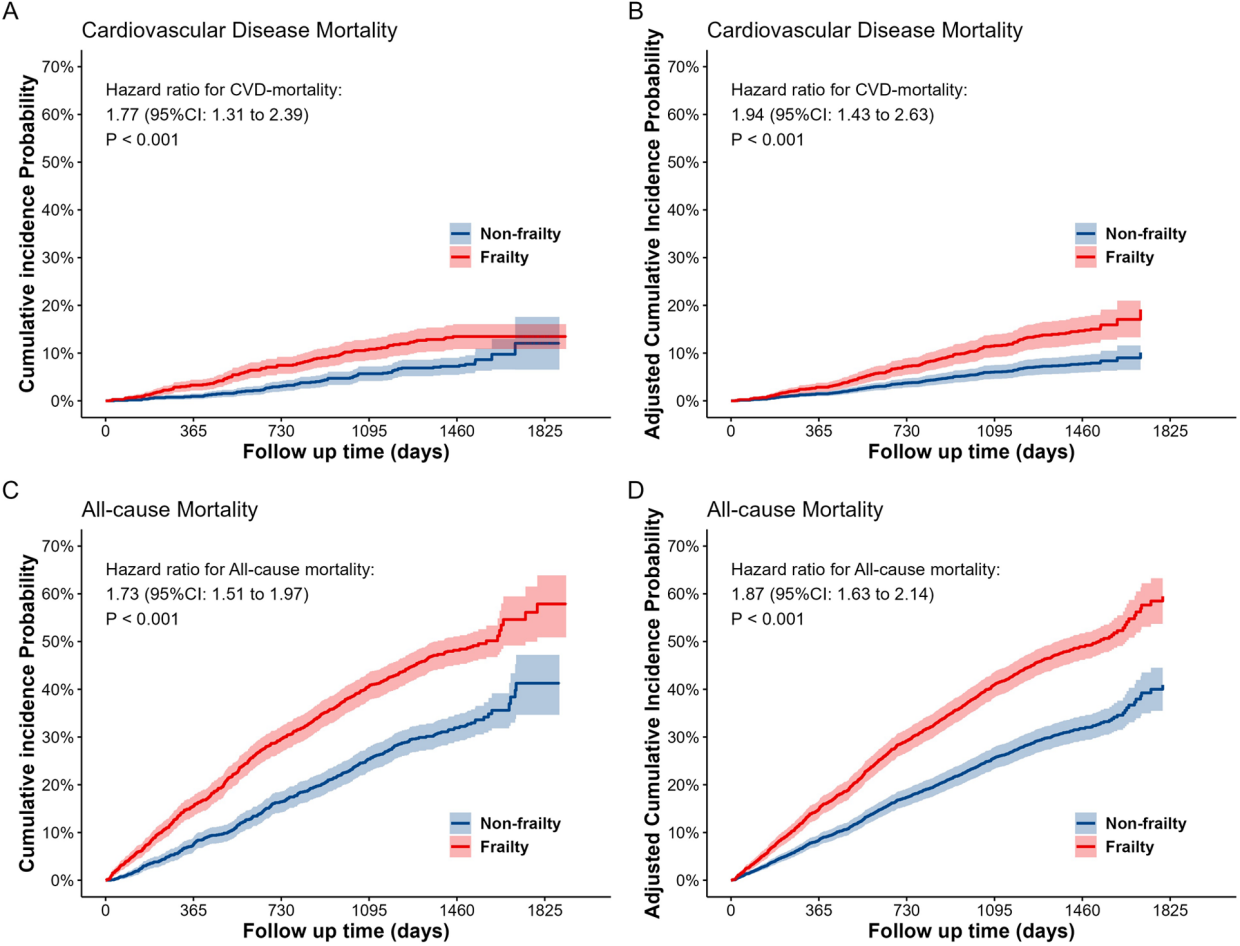
The cumulative incidence probability curves for cardiovascular disease **(A**,**B)** and all-cause **(C**,**D)** mortality between the two groups (frailty and non-frailty) in the matched dataset. **(A)** and **(C)** are the cumulative incidence probability curves without adjusting for covariates, while **(B)** and **(D)** are the cumulative incidence probability curves after adjusting for all covariates.

Frailty is associated with a higher risk of cardiovascular disease and all-cause mortality in older adults. The results of the association analysis are demonstrated in Table 1. In the matched population, model 4 (adjusting for all covariates) demonstrated that the risk of CVD mortality was 1.94 folds (95% CI: 1.43–2.63) higher, and the risk of all-cause mortality was 1.87 folds (95% CI: 1.63–2.14) higher compared with the no-frailty group. Cumulative incidence curves, before and after covariate adjustment, indicated that the risk of CVD and all-cause mortality was higher in the frailty group than in the non-frailty group (see Figure 2). The same results were demonstrated in the unmatched population (see Supplementary Figure S4).

**Table 1:** A Cox analysis of the association between frailty and cardiovascular disease and all-cause mortality.

Model^a^	Frailty group	Original (*n* = 5,084)		Matched (*n* = 2,190)	
		HR (95% CI)	*P*-value	HR (95% CI)	*P*-value
Cardiovascular disease mortality					
Model 1	Non-frailty	1 (Ref.)		1 (Ref.)	
	Frailty	2.94 (2.31–3.74)	<0.001	1.77 (1.31–2.39)	<0.001
Model 2	Non-frailty	1 (Ref.)		1 (Ref.)	
	Frailty	2.41 (1.88–3.09)	<0.001	1.87 (1.38–2.53)	<0.001
Model 3	Non-frailty	1 (Ref.)		1 (Ref.)	
	Frailty	2.25 (1.75–2.90)	<0.001	1.86 (1.37–2.52)	<0.001
Model 4	Non-frailty	1 (Ref.)		1 (Ref.)	
	Frailty	2.28 (1.77–2.95)	<0.001	1.94 (1.43–2.63)	<0.001
All-cause mortality					
Model 1	Non-frailty	1 (Ref.)		1 (Ref.)	
	Frailty	2.56 (2.30–3.84)	<0.001	1.73 (1.51–1.97)	<0.001
Model 2	Non-frailty	1 (Ref.)		1 (Ref.)	
	Frailty	1.96 (1.75–2.18)	<0.001	1.84 (1.61–2.11)	<0.001
Model 3	Non-frailty	1 (Ref.)		1 (Ref.)	
	Frailty	1.87 (1.67–2.09)	<0.001	1.84 (1.61–2.11)	<0.001
Model 4	Non-frailty	1 (Ref.)		1 (Ref.)	
	Frailty	1.85 (1.65–2.07)	<0.001	1.87 (1.63–2.14)	<0.001

BMI, body mass index; HR, hazard ratio; CI, confidence interval; Ref., reference.

^a^ Model: model 1 was unadjusted; model 2 was adjusted for age, sex, ethnicity, residence, co-residence, education, total income, marital status, and BMI; model 3 included additional adjustments for smoking, drinking, exercising, physical labor, social activities, and pension, building upon model 2; model 4 was further adjusted for fruit intake, vegetable intake, edible oil consumption, and meat intake, building upon model 3.

**Figure 3:**
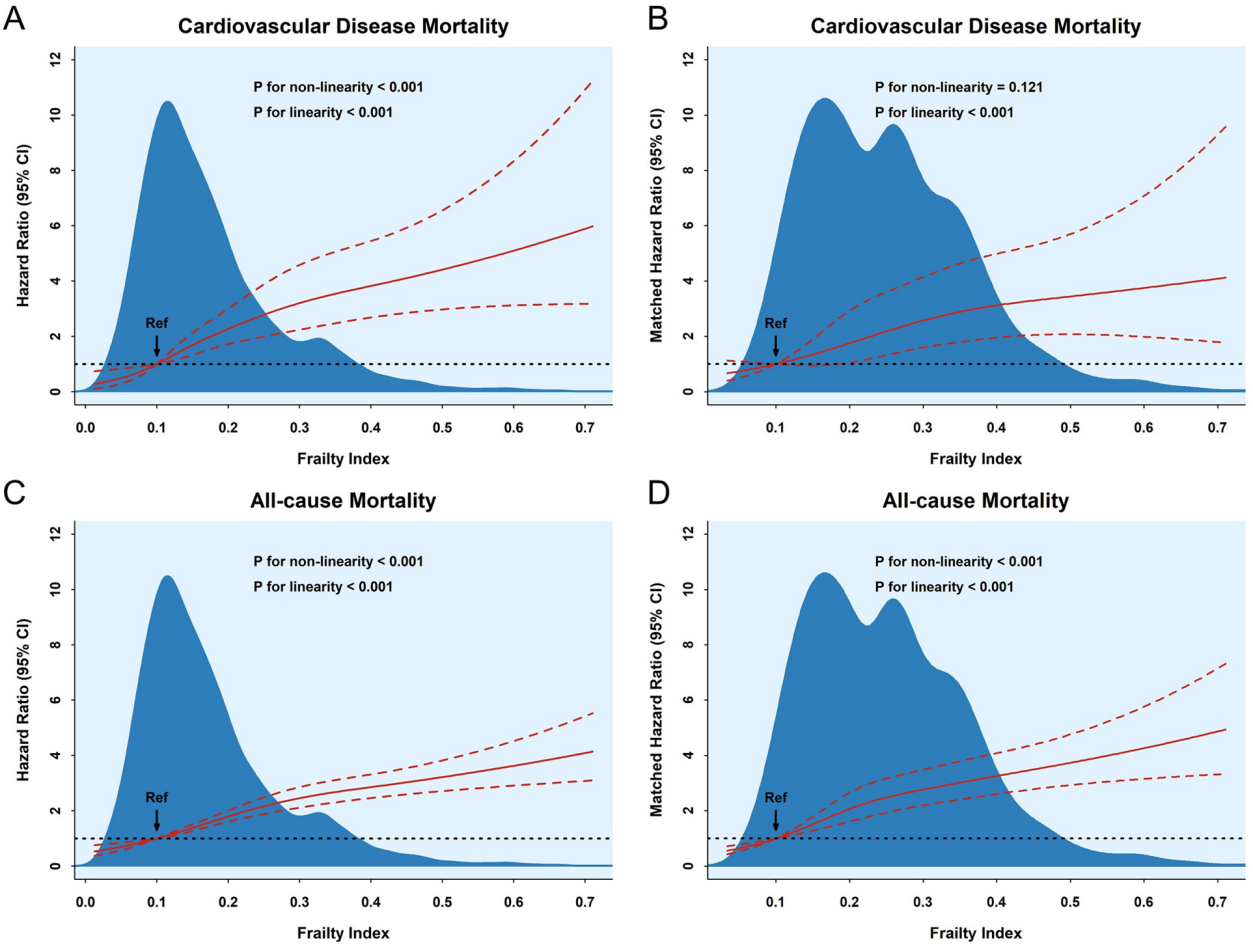
The association between the frailty index and cardiovascular disease **(A**,**B)** and all-cause **(C**,**D)** mortality according to RSC regression, with frailty index = 0.1 as a reference. The hazard ratios and 95% CIs were calculated by adjusting for all covariates.

The dose–response relationship between the frailty index and CVD and all-cause mortality is shown in Figure 3. Analysis of the results in the unmatched dataset revealed a linear relationship between the FI and the risk of developing CVD and all-cause mortality (*P* for linearity < 0.001). However, the results of the analysis of the matching dataset indicated that there was no non-linear relationship between the FI and the risk of incidence of CVD mortality (*P* for non-linearity = 0.121); also, a linear measured response relationship was observed between the FI and both CVD and all-cause mortality (*P* for linearity < 0.001). The RCS results indicated that the risk of incidence of both CVD and all-cause mortality was greater as the FI increased. In addition, there was also a non-linear relationship between age and the risk of all-cause mortality (*P* for non-linearity = 0.0141). The risk of CVD mortality increased with age, but there was no association with BMI. The risk of all-cause mortality increased with age, in contrast to BMI (Supplementary Figure S5).

### 3.3 Association between frailty and cardiovascular disease mortality according to competing risk models

There was a statistically significant difference in CVD mortality between the non-frailty and frailty groups (*P* < 0.01, Supplementary Fig. S6). The results of the cause-of-death competing risk modeling for CVD deaths indicated that frailty was an independent risk factor for the incidence of CVD mortality (Supplementary Table S3). The results of the model after association for all covariates showed that the HR for the frailty group compared with the non-frailty group was 1.94 (95% CI: 1.48–2.53) and 1.62 (95% CI: 1.19–2.20) in the unmatched and matched datasets, respectively. Hence, after considering other death-competing events, frailty remains associated with a higher risk of CVD mortality in older adults.

### 3.4 Subgroup and interaction analysis between the frailty and cardiovascular disease and all-cause mortality

**Figure 4:**
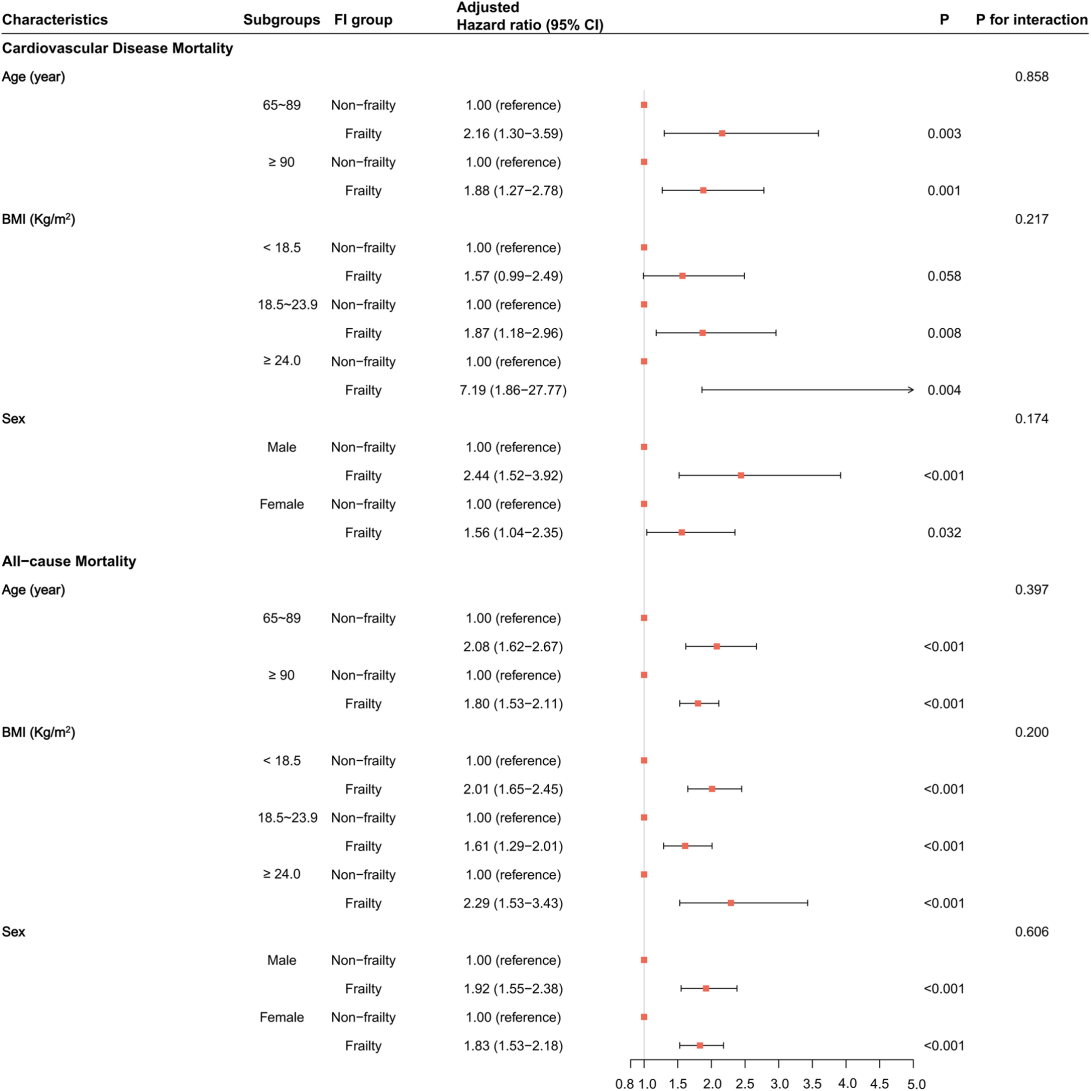
The association of frailty with cardiovascular disease and all-cause mortality in each subgroup in the matched dataset. HRs and their 95% CIs, *P*-values, and interaction *P*-values were calculated by Cox models adjusted for all covariates.

According to the multivariate-adjusted model in the matched dataset, we performed subgroup analyses based on age (65–89 and ≥90 years old), BMI levels (< 18.5, 18.5–23.9, and ≥24.0 kg/m^2^), and sex subgroups (see Figure 4). All subgroups, except the BMI <18.5 subgroup (*P* = 0.058), indicated that frailty was associated with a higher risk of CVD mortality in older adults (*P* < 0.05). However, all subgroups found that frailty was associated with a higher risk of all-cause mortality (*P* < 0.05). Moreover, there was no interaction between subgroup characteristics and frailty (*P* for interaction > 0.05). This suggests that frailty is an independent risk factor for CVD and all-cause mortality.

The RCS model results found no non-linear relationship between the FI and the risk of incidence of CVD mortality in all subgroups (*P* for non-linearity > 0.05, Supplementary Figure S7). However, there was a non-linear association between the FI and the risk of occurrence of all-cause mortality in all subgroups except in the BMI ≥24.0 subgroup (*P* for non-linearity = 0.346, Supplementary Figure S8). With greater FI, the risk of incidence of CVD and all-cause mortality became stronger (*P* for linearity < 0.05). The RCS curves in all subgroups showed more consistent results; that is, greater FI corresponded to a greater risk of incident CVD or all-cause mortality.

### 3.5 Sensitivity analysis

To confirm the robust association between frailty and the risk of incidence of CVD and all-cause mortality, we performed multiple sensitivity analyses. All the results were consistent with primary analyses (Supplementary Table S4). In addition, we also observed that the pre-frailty and frailty groups exhibited similarly increased risk of CVD and all-cause mortality incidence compared with the robust group (Supplementary Table S5). Furthermore, the results of the competing risk analysis in the complete matched datasets were also consistent with the primary results (Supplementary Table S6).

## 4 Discussion

This is a study to observe the association between frailty and the incidence of CVD and all-cause mortality among community-based older adults in China. Our results found that frail older adults were more likely to experience CVD and all-cause mortality compared with the non-frail ones. There was a dose–response relationship for this association; that is, the risk of CVD and all-cause mortality became stronger with a greater frailty index. Individuals with a smaller FI, such as those between 0.1 and 0.25 (often considered pre-frailty), have a smaller risk of death compared with those with a greater FI (FI ≥ 0.25), especially in robust individuals (FI < 0.1). Therefore, this implies that reversing or delaying a frail status can contribute to reducing the risk of death in older adults. Subsequently, subgroup and multiple sensitivity analyses were performed to verify the stability of the findings obtained, and the results demonstrated consistency.

Our study found the prevalence of frailty to be 21.9% (1,111/5,084), which is consistent with the findings of most previous studies (18, 33). In addition, the mortality rate in this study was higher than in a previous study conducted in China (24). In that study, the all-cause mortality rate per 1,000 person-years was 54.6. The mortality rates for ischemic heart disease and cerebrovascular disease per 1,000 person-years were 9.6 and 12.9, respectively, which we believe may be because our study population consisted of older adults aged 65 years or older, compared with adults aged 30–79 years in their study. The reason why older adults have a higher mortality rate than younger adults is because they are more susceptible to a variety of diseases. Biologically, aging is the result of a gradual accumulation of various molecular and cellular damages over time, which contribute to a gradual decline in physical abilities and an increasing risk of disease and, ultimately, death.

Frailty has received much attention in recent years as one of the most commonly used indicators for evaluating the biological age of an individual. Several studies have reported the association between frailty and CVD mortality or other mortality. Fan et al. found that frailty increased the risk of all-cause mortality, ischemic heart disease, cerebrovascular disease, cancer, and other deaths in a subgroup of individuals aged 65 years or older, as well as in those younger than 65 years (24). Furthermore, a review study by Ekram et al. showed that using three scales for assessing frailty, the estimated risk ratio between frailty and all-cause mortality was 2.34 (95% CI: 1.77–3.09), and their association did not differ between ages (34). The results of a meta-analysis suggested that the FI was a significant predictor of mortality (15). Each 0.01 increase in the FI was associated with a 4% increase in the mortality risk. Matsuo et al. investigated the association between frailty and the risk of mortality among people aged 65 years or older who underwent health checkups in Japan and found that, compared with the healthy group, the pre-frailty group and the frailty group had an increased risk of all-cause mortality, cardiovascular disease, respiratory disease, and cancer mortality (33). Shi et al. found a positive correlation between an increase in FI over a 1-year period and an increased risk of mortality (35). In summary, the above studies confirm the association between frailty and CVD and all-cause mortality. This is consistent with the main finding of the present study.

Frail older adults are more susceptible to cardiovascular events and have significantly higher mortality (36). This association may be due to the combined effect of multiple physiologic and pathologic factors. The relationship between frailty and CVD mortality and all-cause mortality in older adults is complex and diverse. With increasing age, the cardiovascular system undergoes multiple changes, including atherosclerosis, decreased vascular elasticity, and dysregulation of the autonomic nervous system (37–39). These changes may be more pronounced in the frail state, resulting in older adults being more vulnerable to cardiovascular risk factors. Research suggests that the frail state may be associated with a decline in the cardiovascular system (40). These physiologic changes may increase the risk of cardiovascular disease in older adults. In addition, frailty may indirectly affect the development and progression of cardiovascular disease by affecting the overall resistance, immune system function, and inflammatory status of older adults (41, 42). Therefore, frailty consistently increases the risk of developing cardiovascular mortality. The trend toward non-linearity between FI and all-cause mortality may also be related to physiologic reserve in older adults, the interplay of multiple chronic conditions, and other factors that may influence mortality risk. At lower levels of the FI, several physiologic and health problems may begin to manifest themselves, resulting in a dramatic increase in mortality risk. However, once a certain level of frailty is reached, several physiological adaptive mechanisms may have been triggered, leading to a slowing down of the increase in mortality risk. This view is supported by the theory of physiological redundancy (27, 43). This is why the results of our subgroup analyses found that frailty was less associated with the risk of cardiovascular and all-cause mortality in advanced age (≥90 years) than in other older adults (65–89 years).

Our study revealed several findings of interest. Consumption of animal oils, such as lard, was associated with a lower risk of CVD mortality (HR: 0.53, 95% CI: 0.34–0.84) in older adults compared with the consumption of vegetable oils. This is in agreement with the findings of Wang et al. (44). However, the biological mechanisms associated with it are not clear. This may be related to the unique dietary habits of older Chinese adults. We also found that being underweight was associated with a higher risk of all-cause mortality in older adults (HR: 1.19, 95% CI: 1.06–1.33) in older adults. Several studies have suggested that being moderately underweight may be associated with a lower risk of all-cause mortality, especially in older adults (45). Underweight older people may suffer from malnutrition, which can lead to a decline in their immune function and reduce their disease resistance. In addition, a prospective research study confirmed that being underweight is associated with a higher risk of stroke, myocardial infarction, and death (46).

There are several advantages to our study. First, the study has a large sample size based on a national survey, which is extremely representative of older Chinese adults. It is also a prospective cohort study, which found a strong causal effect of frailty on the risk of CVD and all-cause mortality. Second, we also used propensity score methods to control for potential confounders, alongside multiple sensitivity analyses and competing risk analyses, ensuring that the findings were extremely reliable.

### 4.1 Limitations

This study has several notable limitations. First, the population in this study was mainly older Chinese adults, and the findings may not apply to other countries or regions. Of course, several other studies agree with the main findings of our study (15). Second, we did not include those under 65 years of age, who were also found to have a higher prevalence of frailty (47). Third, the frail state is unstable and worsens or reverses with age. Due to the restrictions of available data, this study only considered the frailty state at baseline. Finally, the present study was observational, and it is difficult to avoid the presence of unknown confounders interfering with the study, even though propensity score methods were used.

### 4.2 Future directions

Future studies should examine the association between dynamically changing frailty status and the risk of CVD or multiple other causes of mortality using long-term follow-up data from larger older populations. In addition, measures to control for potential confounders in observational studies are needed.

## 5 Conclusion

Frailty in older adults is associated with a higher risk of cardiovascular disease and all-cause mortality. Even when other competing events for death were considered, frailty remained associated with higher CVD mortality. In addition, the risk of cardiovascular disease and all-cause mortality increased as the frailty index increased. Our findings could help prevent deaths in frail older adults, and improving their lifestyles could help reduce mortality. Further longitudinal studies of frailty dynamics in older populations are necessary.

## Data Availability

The raw data supporting the conclusions of this article will be made available by the authors without undue reservation.
