# Supplementary material for: Association of frailty with cardiovascular and all-cause mortality in community-dwelling older adults: insights from the Chinese longitudinal healthy longevity survey
Source: Front Cardiovasc Med. 2024 Dec 23;11:1499099. doi: 10.3389/fcvm.2024.1499099 (PMC11701156; doi:10.3389/fcvm.2024.1499099)
Supplement: Supplementary file 1 [file Datasheet1.pdf]

Association of Frailty with Cardiovascular and All-Cause Mortality in Community-Dwelling Older Adults: Insights from the Chinese Longitudinal Healthy Longevity Survey

Supplementary files

Legend:
Table S1 The health deficits used to construct the frailty index.
Table S2 Characteristics of study participants according to the frailty status.
Table S3 Competing risk analysis of the association between frailty and cardiovascular disease mortality.
Table S4 Association between frailty and cardiovascular disease and all-cause mortality in the sensitive analysis.
Table S5 Association between frailty and cardiovascular disease and all-cause mortality in the sensitive analysis, frail state as a multi-categorical variable (robust, pre-frailty, and frailty).
Table S6 Competing risk analyses between frailty and cardiovascular disease mortality in the complete dataset after multiple imputation.

Figure S1 The cumulative incidence probability curves for cardiovascular disease and all-cause mortality for the total population.
Figure S2 Plots of conditional probability distributions for each individual between the two groups (frailty and non-frailty) in the original dataset (left) and post-matching (right) dataset based on propensity score.
Figure S3 Balance test of the respective variables between the two groups (frailty and non-frailty) after matching based on the propensity score. Equilibrium was considered better with an SMD < 0.1.
Figure S4 The Cumulative incidence probability curves for cardiovascular disease (A, B) and all-cause (C, D) mortality between the two groups (frailty and non-frailty) in the original dataset. A and C are the cumulative incidence probability curves without adjusting for covariates, and B and D are the cumulative incidence probability curves after adjusting for all covariates.
Figure S5 Association between age (A, B) or BMI (C, D) and cardiovascular disease (A, C) and all-cause (B, D) mortality according to RSC regression, with age = 80 years and BMI = 24 Kg/m^2 as reference. The hazard ratio and 95% CI were calculated by adjusting for all covariates. The solid red line indicates the RCS curve and the dashed red line indicates the 95% CI.
Figure S6 The cumulative incidence probability curves for cardiovascular disease mortality using competing risk models. All other cause mortality events were used as

competing risk events.

Figure S7 Association between the frailty index and cardiovascular disease mortality according to RSC regression in BMI subgroups (A, B, C), age subgroups (D, E), and sex subgroups (F, G), with frailty index = 0.1 as reference. The hazard ratio and 95% CI were calculated by adjusting for all covariates. The solid red line indicates the RCS curve and the dashed red line indicates the 95% CI.

Figure S8 Association between the frailty index and all-cause disease mortality according to RSC regression in BMI subgroups (A, B, C), age subgroups (D, E), and sex subgroups (F, G), with frailty index = 0.1 as reference. The hazard ratio and 95% CI were calculated by adjusting for all covariates. The solid red line indicates the RCS curve and the dashed red line indicates the 95% CI.

Table S1 The health deficits used to construct the frailty index.

	Variable name	Response/code
1	Self-reported health	very good=0; good=0.25; average=0.5; bad=0.75; very bad=1
2	Look on the bright side of things	always=0; often=0.25; sometimes=0.5; seldom=0.75; never=1
3	Keep my belongings neat and clean	always=0; often=0.25; sometimes=0.5; seldom=0.75; never=1
4	Feel fearful or anxious	always=1; often=0.75; sometimes=0.5; seldom=0.25; never=0
5	Feel lonely and isolated	always=1; often=0.75; sometimes=0.5; seldom=0.25; never=0
6	Feel useless because of age	always=1; often=0.75; sometimes=0.5; seldom=0.25; never=0
7	Make own decisions	always=0; often=0.25; sometimes=0.5; seldom=0.75; never=1
8	Do housework at present	almost every day=0; not daily, but at least once a week=0.5; not weekly, but at least once a month=0.5; not monthly, but sometimes=0.5; never=1
9	Feeding	without assistance=0; some assistance=0.5; a lot of assistance=1
10	Bathing	without assistance=0; assistance with 1 body part=0.5; assistance with >1 body part=1
11	Dressing	without assistance=0; some assistance=0.5; a lot of assistance=1
12	Toileting	without assistance=0; some assistance=0.5; a lot of assistance=1
13	Indoor activities	without assistance=0; some assistance=0.5; a lot of assistance=1
14	Continence	able=0; occasional incontinence=0.5; frequent incontinence=1
15	Cognitive function (MMSE score)	≥24 = 0; 18-23 = 0.5; <18 = 1
16	Sleep quality	very good=0; good=0.25; average=0.5; bad=0.75; very bad=1
17	Visual function	can see and distinguish the break in the circle=0; can see but not distinguish the break in the circle=0.33; cannot see=0.67; blind=1
18	Hearing ability	can hear without a hearing aid=0; can hear with a hearing aid=0.33; partly deaf, despite using a hearing aid=0.67; deaf=1
19	Hand behind neck	right hand=0.5; left hand=0.5; both hands=0; neither hand=1
20	Hand behind lower back	right hand=0.5; left hand=0.5; both hands=0; neither hand=1
21	Hold-up arms	only left arm=0.5; only right arm=0.5; two arms=0; neither arm=1
22	Cardiac rhythm	regular=0; irregular=1
23	Able to use chopsticks to eat	yes=0; no=1
24	Able to stand up from sitting	yes, without using hands=0; yes, using hands=0.5; no=1
25	Able to pick up a book from the floor	yes, standing=0; yes, sitting=0.5; no=1
26	Number of steps used to turn around a 360 degree turn without help	≤4=0; >4=0.5; cannot turn around=1
27	Number of times suffering from serious illness in the past two years	0=0; 1=0.5; >1=1
28	Interviewer rated-health	surprisingly healthy=0; relatively healthy=0.33; moderately unhealthy=0.67; very unhealthy=1

29	Hypertension	yes=1; no=0
30	Diabetes	yes=1; no=0
31	Heart disease	yes=1; no=0
32	Stroke or cerebrovascular disease	yes=1; no=0
33	Bronchitis, emphysema, pneumonia, asthma	yes=1; no=0
34	Tuberculosis	yes=1; no=0
35	Cancer	yes=1; no=0
36	Gastric or duodenal ulcer	yes=1; no=0
37	Parkinson’s disease	yes=1; no=0
38	Bedsores	yes=1; no=0
39	Dementia	yes=1; no=0

MMSE = Mini-Mental State Examination (MMSE).

Table S2 Characteristics of study participants according to the frailty status.

Characteristics	Original					Matched			
	Overall<br>*n* = 5084	Non-frailty<br>*n* = 3973	Frailty<br>*n* = 1111	P value	SMD	Non-frailty<br>*n* = 1095	Frailty<br>*n* = 1095	P value	SMD
Age (year), n (%)									
65-79	1773 (34.9)	1551 (39.0)	222 (20.0)	<0.001	0.555	215 (19.6)	222 (20.3)	0.834	0.026
80-89	1668 (32.8)	1351 (34.0)	317 (28.5)			308 (28.1)	315 (28.8)		
≥ 90	1643 (32.3)	1071 (27.0)	572 (51.5)			572 (52.2)	558 (51.0)		
BMI (Kg/m^2), n (%)									
Normal	2362 (46.5)	1913 (48.2)	449 (40.4)	<0.001	0.218	466 (42.6)	447 (40.8)	0.569	0.061
Underweight	1681 (33.1)	1233 (31.0)	448 (40.3)			443 (40.5)	437 (39.9)		
Overweight	692 (13.6)	566 (14.2)	126 (11.3)			111 (10.1)	125 (11.4)		
Obese	349 (6.9)	261 (6.6)	88 (7.9)			75 (6.8)	86 (7.9)		
Sex, n (%)									
Male	2391 (47.0)	1953 (49.2)	438 (39.4)	<0.001	0.197	436 (39.8)	435 (39.7)	0.999	0.002
Female	2693 (53.0)	2020 (50.8)	673 (60.6)			659 (60.2)	660 (60.3)		
Ethnic, n (%)									
Han	4697 (92.4)	3636 (91.5)	1061 (95.5)	<0.001	0.162	1037 (94.7)	1045 (95.4)	0.490	0.034
Other	387 (7.6)	337 (8.5)	50 (4.5)			58 (5.3)	50 (4.6)		
Residence, n (%)									
City	784 (15.4)	595 (15.0)	189 (17.0)	0.022	0.094	177 (16.2)	185 (16.9)	0.513	0.049
Town	1696 (33.4)	1303 (32.8)	393 (35.4)			414 (37.8)	388 (35.4)		
Rural	2604 (51.2)	2075 (52.2)	529 (47.6)			504 (46.0)	522 (47.7)		
Co-residence, n (%)									
Family members	4048 (79.6)	3133 (78.9)	915 (82.4)	<0.001	0.243	914 (83.5)	903 (82.5)	0.819	0.027
Alone	924 (18.2)	779 (19.6)	145 (13.1)			136 (12.4)	145 (13.2)		

Nursing home	112 (2.2)	61 (1.5)	51 (4.6)			45 (4.1)	47 (4.3)		
Education, n (%)									
Uneducated	2814 (55.4)	2089 (52.6)	725 (65.3)	<0.001	0.263	718 (65.6)	711 (64.9)	0.781	0.030
Primary	1662 (32.7)	1370 (34.5)	292 (26.3)			292 (26.7)	290 (26.5)		
Junior and above	608 (12.0)	514 (12.9)	94 (8.5)			85 (7.8)	94 (8.6)		
Total income (CNY), n (%)									
Low	2491 (49.0)	1968 (49.5)	523 (47.1)	0.157	0.049	488 (44.6)	515 (47.0)	0.265	0.050
High	2593 (51.0)	2005 (50.5)	588 (52.9)			607 (55.4)	580 (53.0)		
Marital status, n (%)									
Married	2108 (41.5)	1786 (45.0)	322 (29.0)	<0.001	0.335	320 (29.2)	322 (29.4)	0.963	0.004
Other [a]	2976 (58.5)	2187 (55.0)	789 (71.0)			775 (70.8)	773 (70.6)		
Smoking, n (%)									
Never	3450 (67.9)	2631 (66.2)	819 (73.7)	<0.001	0.244	811 (74.1)	805 (73.5)	0.890	0.021
Former	729 (14.3)	559 (14.1)	170 (15.3)			160 (14.6)	168 (15.3)		
Present	905 (17.8)	783 (19.7)	122 (11.0)			124 (11.3)	122 (11.1)		
Drinking, n (%)									
Never	3737 (73.5)	2863 (72.1)	874 (78.7)	<0.001	0.291	871 (79.5)	862 (78.7)	0.888	0.021
Former	514 (10.1)	375 (9.4)	139 (12.5)			131 (12.0)	135 (12.3)		
Present	833 (16.4)	735 (18.5)	98 (8.8)			93 (8.5)	98 (8.9)		
Exercising, n (%)									
No	3889 (76.5)	2915 (73.4)	974 (87.7)	<0.001	0.367	954 (87.1)	958 (87.5)	0.847	0.011
Yes	1195 (23.5)	1058 (26.6)	137 (12.3)			141 (12.9)	137 (12.5)		
Physical labor, n (%)									
No	931 (18.3)	683 (17.2)	248 (22.3)	<0.001	0.129	245 (22.4)	245 (22.4)	0.999	<0.001
Yes	4153 (81.7)	3290 (82.8)	863 (77.7)			850 (77.6)	850 (77.6)		
Social activities, n (%)									

Never	4296 (84.5)	3276 (82.5)	1020 (91.8)	<0.001	0.282	996 (91.0)	1005 (91.8)	0.716	0.035
Sometimes	412 (8.1)	364 (9.2)	48 (4.3)			56 (5.1)	48 (4.4)		
Often	376 (7.4)	333 (8.4)	43 (3.9)			43 (3.9)	42 (3.8)		
Pension, n (%)									
No	4004 (78.8)	3122 (78.6)	882 (79.4)	0.589	0.020	899 (82.1)	867 (79.2)	0.094	0.074
Yes	1080 (21.2)	851 (21.4)	229 (20.6)			196 (17.9)	228 (20.8)		
Fruits intake, n (%)									
Everyday	712 (14.0)	567 (14.3)	145 (13.1)	<0.001	0.191	139 (12.7)	145 (13.2)	0.983	0.018
Often	1342 (26.4)	1113 (28.0)	229 (20.6)			233 (21.3)	229 (20.9)		
Occasionally	1711 (33.7)	1302 (32.8)	409 (36.8)			404 (36.9)	404 (36.9)		
Never	1319 (25.9)	991 (24.9)	328 (29.5)			319 (29.1)	317 (28.9)		
Vegetables intake, n (%)									
Everyday	2931 (57.7)	2373 (59.7)	558 (50.2)	<0.001	0.270	577 (52.7)	556 (50.8)	0.830	0.040
Often	1603 (31.5)	1236 (31.1)	367 (33.0)			349 (31.9)	365 (33.3)		
Occasionally	378 (7.4)	268 (6.7)	110 (9.9)			108 (9.9)	109 (10.0)		
Never	172 (3.4)	96 (2.4)	76 (6.8)			61 (5.6)	65 (5.9)		
Edible oil, n (%)									
Vegetable oil	4463 (87.8)	3453 (86.9)	1010 (90.9)	<0.001	0.128	1007 (92.0)	994 (90.8)	0.361	0.042
Animal oil	621 (12.2)	520 (13.1)	101 (9.1)			88 (8.0)	101 (9.2)		
Meat intake, n (%)									
Everyday	1960 (38.6)	1563 (39.3)	397 (35.7)	<0.001	0.145	395 (36.1)	391 (35.7)	0.878	0.035
Per week	2093 (41.2)	1642 (41.3)	451 (40.6)			455 (41.6)	449 (41.0)		
Occasionally	667 (13.1)	516 (13.0)	151 (13.6)			152 (13.9)	151 (13.8)		
Never	364 (7.2)	252 (6.3)	112 (10.1)			93 (8.5)	104 (9.5)		

[a] other: including unmarried, divorced, separate and widowed. BMI = body mass index; SMD = standardized mean difference, the SMD < 0.1 was considered better for between-group balance.

Table S3 Competing risk analysis of the association between frailty and cardiovascular disease mortality.

Model [a]	Frailty group	**Original** ***n* = 5084** HR (95% CI)	P value	**Matched** ***n* = 2190** HR (95% CI)	P value
Model 1	Non-frailty	1 (Ref.)		1 (Ref.)	
	Frailty	2.47 (1.94-3.13)	<0.001	1.56 (1.16-2.11)	0.004
Model 2	Non-frailty	1 (Ref.)		1 (Ref.)	
	Frailty	2.06 (1.60-2.66)	<0.001	1.60 (1.18-2.16)	0.003
Model 3	Non-frailty	1 (Ref.)		1 (Ref.)	
	Frailty	1.94 (1.49-2.52)	<0.001	1.58 (1.16-2.14)	0.004
Model 4	Non-frailty	1 (Ref.)		1 (Ref.)	
	Frailty	1.94 (1.48-2.53)	<0.001	1.62 (1.19-2.20)	0.002

[a] Model: model 1 was unadjusted; model 2 was adjusting for age, sex, ethnic, residence, co-residence, education, total income, marital status, and BMI; model 3 with additional adjustment for smoking, drinking, exercising, physical labor, social activities, and pension upon model 2; model 4 with additional adjustment for fruits intake, vegetables intake, edible oil, and meat intake upon model 3. BMI = body mass index; HR = hazard ratio; CI = confidence interval; Ref. = reference.

Table S4 Association between frailty and cardiovascular disease and all-cause mortality in the sensitive analysis.

Model [a]	Frailty group	**IPTW-Cox Regression** ***n* = 5084**		**Multiple imputation** ***n* = 6842**		**Multiple imputation-Matched** ***n* = 3212-3234**	
		HR (95% CI)	P value	HR (95% CI)	P value	HR (95% CI)	P value
**Cardiovascular Disease Mortality**							
Model 1	Non-frailty	1 (Ref.)		1 (Ref.)		1 (Ref.)	
	Frailty	2.17 (1.64-2.86)	<0.001	3.38 (2.79-4.09)	<0.001	2.31 (1.73-3.08)	<0.001
Model 2	Non-frailty	1 (Ref.)		1 (Ref.)		1 (Ref.)	
	Frailty	2.28 (1.74-2.99)	<0.001	2.76 (2.26-3.38)	<0.001	2.41 (1.80-3.22)	<0.001
Model 3	Non-frailty	1 (Ref.)		1 (Ref.)		1 (Ref.)	
	Frailty	2.29 (1.74-3.02)	<0.001	2.58 (2.11-3.17)	<0.001	2.40 (1.80-3.22)	<0.001
Model 4	Non-frailty	1 (Ref.)		1 (Ref.)		1 (Ref.)	
	Frailty	2.36 (1.79-3.09)	<0.001	2.56 (2.08-3.15)	<0.001	2.47 (1.85-3.31)	<0.001
**All-cause Mortality**							
Model 1	Non-frailty	1 (Ref.)		1 (Ref.)		1 (Ref.)	
	Frailty	1.76 (1.55-2.01)	<0.001	2.76 (2.53-3.01)	<0.001	1.80 (1.60-2.03)	<0.001
Model 2	Non-frailty	1 (Ref.)		1 (Ref.)		1 (Ref.)	
	Frailty	1.84 (1.63-2.09)	<0.001	2.06 (1.88-2.26)	<0.001	1.88 (1.67-2.12)	<0.001
Model 3	Non-frailty	1 (Ref.)		1 (Ref.)		1 (Ref.)	
	Frailty	1.86 (1.64-2.10)	<0.001	1.98 (1.80-2.17)	<0.001	1.88 (1.67-2.12)	<0.001
Model 4	Non-frailty	1 (Ref.)		1 (Ref.)		1 (Ref.)	
	Frailty	1.88 (1.66-2.13)	<0.001	1.96 (1.78-2.15)	<0.001	1.89 (1.68-2.14)	<0.001

[a] Model: model 1 was unadjusted; model 2 was adjusting for age, sex, ethnic, residence, co-residence, education, total income, marital status, and BMI; model 3 with additional adjustment for smoking, drinking, exercising, physical labor, social activities, and pension upon model 2; model 4 with additional adjustment for fruits intake, vegetables intake, grease, and meat intake upon model 3. BMI = body mass index; HR = hazard ratio; CI = confidence interval; Ref. = reference.

Table S5 Association between frailty and cardiovascular disease and all-cause mortality in the sensitive analysis, frail state as a multi-categorical variable (robust, pre-frailty, and frailty).

Model [a]	Frailty group	Original *n* = 5084		Multiple imputation *n* = 6842	
		HR (95% CI)	P value	HR (95% CI)	P value
**Cardiovascular Disease Mortality**					
Model 1	Robust	1 (Ref.)		1 (Ref.)	
	Pre-frailty	3.89 (2.33-6.52)	<0.001	4.31 (2.69-6.89)	<0.001
	Frailty	8.98 (5.32-15.16)	<0.001	11.37 (7.10-18.21)	<0.001
Model 2	Robust	1 (Ref.)		1 (Ref.)	
	Pre-frailty	3.16 (1.88-5.31)	<0.001	3.38 (2.10-5.43)	<0.001
	Frailty	6.46 (3.78-11.05)	<0.001	7.99 (4.93-12.94)	<0.001
Model 3	Robust	1 (Ref.)		1 (Ref.)	
	Pre-frailty	3.02 (1.79-5.09)	<0.001	3.19 (1.98-5.13)	<0.001
	Frailty	5.91 (3.43-10.17)	<0.001	7.20 (4.43-11.72)	<0.001
Model 4	Robust	1 (Ref.)		1 (Ref.)	
	Pre-frailty	3.01 (1.79-5.04)	<0.001	3.16 (1.97-5.09)	<0.001
	Frailty	6.00 (3.47-10.37)	<0.001	7.12 (4.36-11.62)	<0.001
**All-cause Mortality**					
Model 1	Robust	1 (Ref.)		1 (Ref.)	
	Pre-frailty	2.32 (1.94-2.77)	<0.001	2.45 (2.08-2.88)	<0.001
	Frailty	4.95 (4.12-5.96)	<0.001	5.62 (4.78-6.62)	<0.001
Model 2	Robust	1 (Ref.)		1 (Ref.)	
	Pre-frailty	1.75 (1.46-2.10)	<0.001	1.79 (1.52-2.11)	<0.001
	Frailty	3.13 (2.58-3.80)	<0.001	3.38 (2.85-4.01)	<0.001
Model 3	Robust	1 (Ref.)		1 (Ref.)	
	Pre-frailty	1.69 (1.41-2.03)	<0.001	1.71 (1.45-2.02)	<0.001
	Frailty	2.92 (2.40-3.55)	<0.001	3.15 (2.65-3.74)	<0.001
Model 4	Robust	1 (Ref.)		1 (Ref.)	
	Pre-frailty	1.68 (1.40-2.02)	<0.001	1.71 (1.45-2.02)	<0.001
	Frailty	2.88 (2.36-3.51)	<0.001	3.12 (2.62-3.72)	<0.001

[a] Model: model 1 was unadjusted; model 2 was adjusting for age, sex, ethnic, residence, co-residence, education, total income, marital status, and BMI; model 3 with additional adjustment for smoking, drinking, exercising, physical labor, social activities, and pension upon model 2; model 4 with additional adjustment for fruits intake, vegetables intake, grease, and meat intake upon model 3. BMI = body mass index; HR = hazard ratio; CI = confidence interval; Ref. = reference.

Table S6 Competing risk analyses between frailty and cardiovascular disease mortality in the complete dataset after multiple imputation.

Model [a]	Frailty group	**Multiple imputation-Matched** ***n*** **= 3136-3314**	
		HR (95% CI)	P value
Model 1	Non-frailty	1 (Ref.)	
	Frailty	1.95 (1.53-2.49)	<0.001
Model 2	Non-frailty	1 (Ref.)	
	Frailty	1.95 (1.53-2.49)	<0.001
Model 3	Non-frailty	1 (Ref.)	
	Frailty	1.94 (1.51-2.48)	<0.001
Model 4	Non-frailty	1 (Ref.)	
	Frailty	1.96 (1.53-2.51)	<0.001

[a] Model: model 1 was unadjusted; model 2 was adjusting for age, sex, ethnic, residence, co-residence, education, total income, marital status, and BMI; model 3 with additional adjustment for smoking, drinking, exercising, physical labor, social activities, and pension upon model 2; model 4 with additional adjustment for fruits intake, vegetables intake, grease, and meat intake upon model 3. BMI = body mass index; HR = hazard ratio; CI = confidence interval; Ref. = reference.

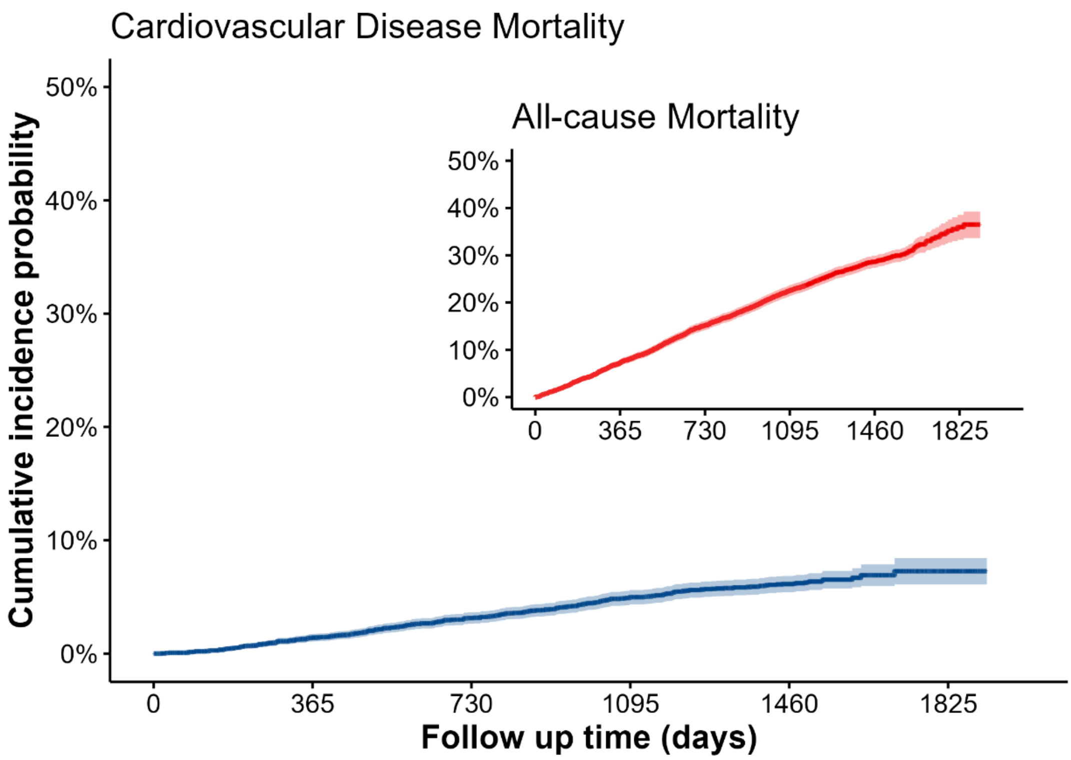

Figure S1 The cumulative incidence probability curves for cardiovascular disease and all-cause mortality for the total population.

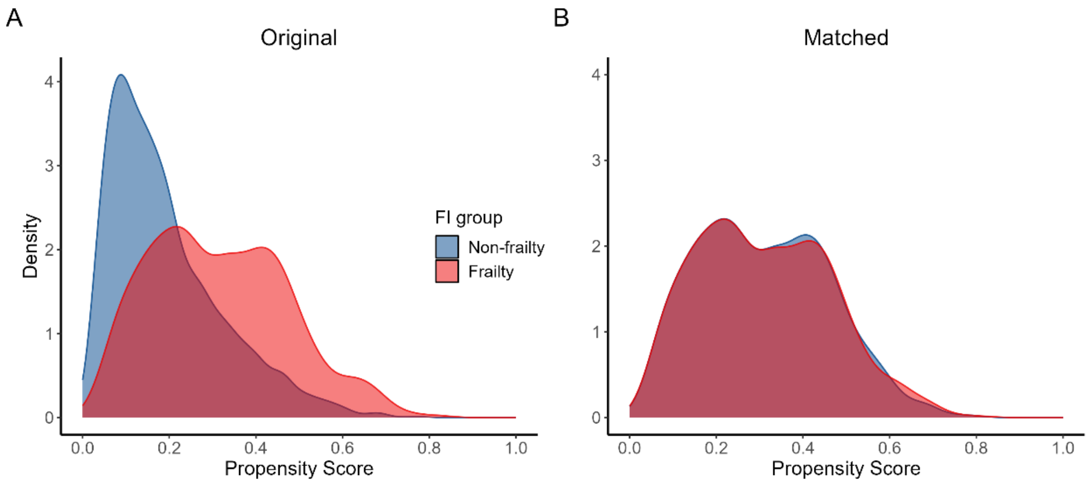

Figure S2 Plots of conditional probability distributions for each individual between two groups (frailty and non-frailty) in the original dataset (left) and post-matching (right) dataset based on propensity score.

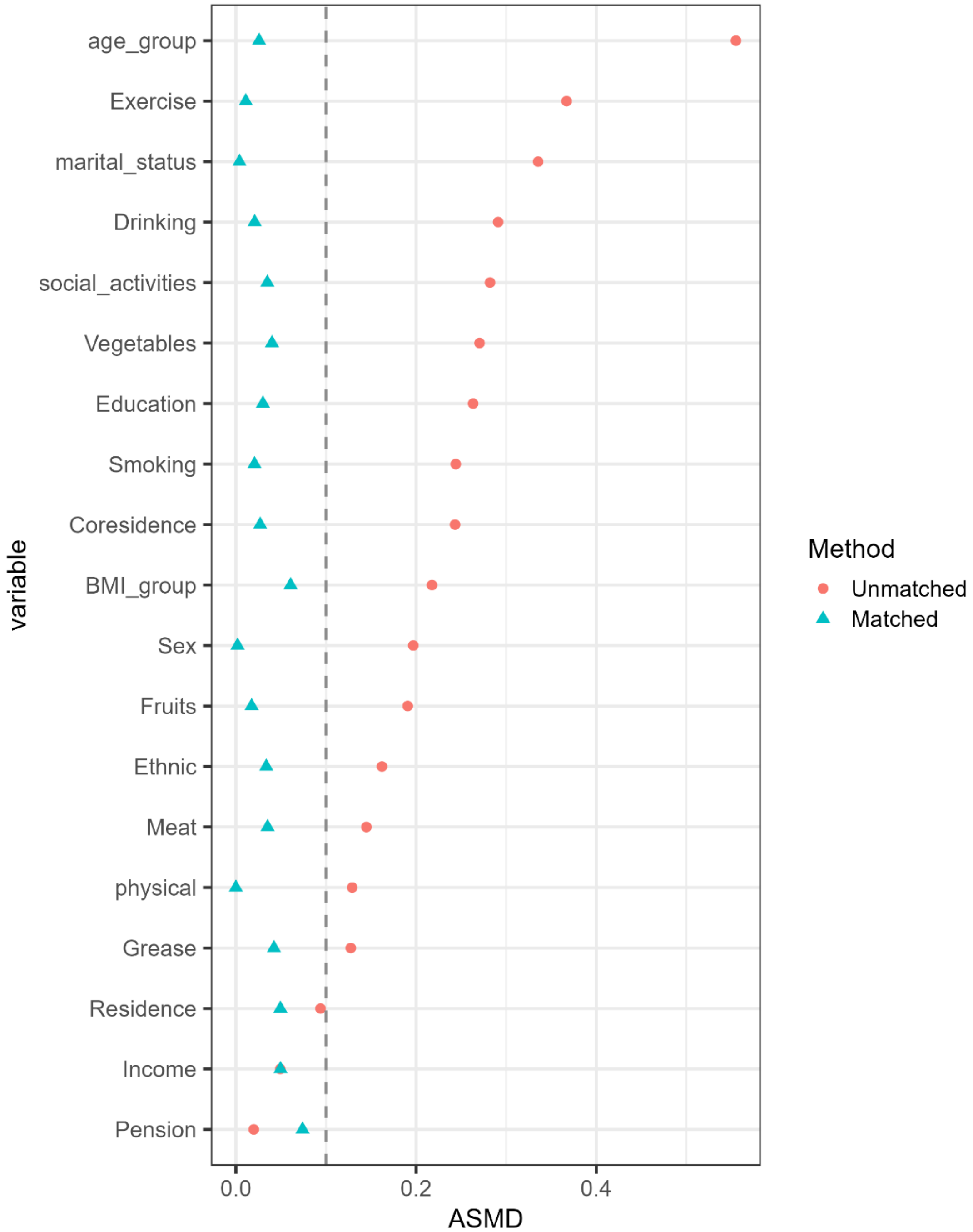

Figure S3 Balance test of the respective variables between the two groups (frailty and non-frailty) after matching based on the propensity score. Equilibrium was considered better with an SMD < 0.1.

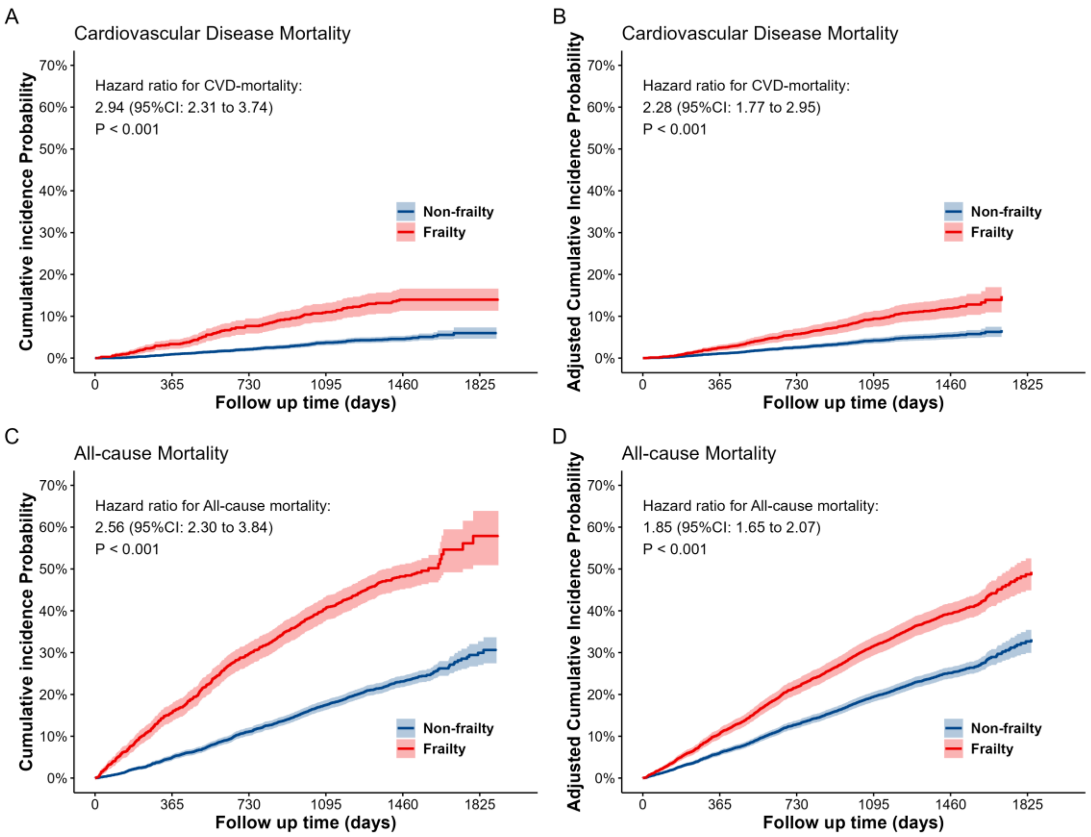

Figure S4 The Cumulative incidence probability curves for cardiovascular disease (A, B) and all-cause (C, D) mortality between the two groups (frailty and non-frailty) in the original dataset. A and C are the cumulative incidence probability curves without adjusting for covariates, and B and D are the cumulative incidence probability curves after adjusting for all covariates.

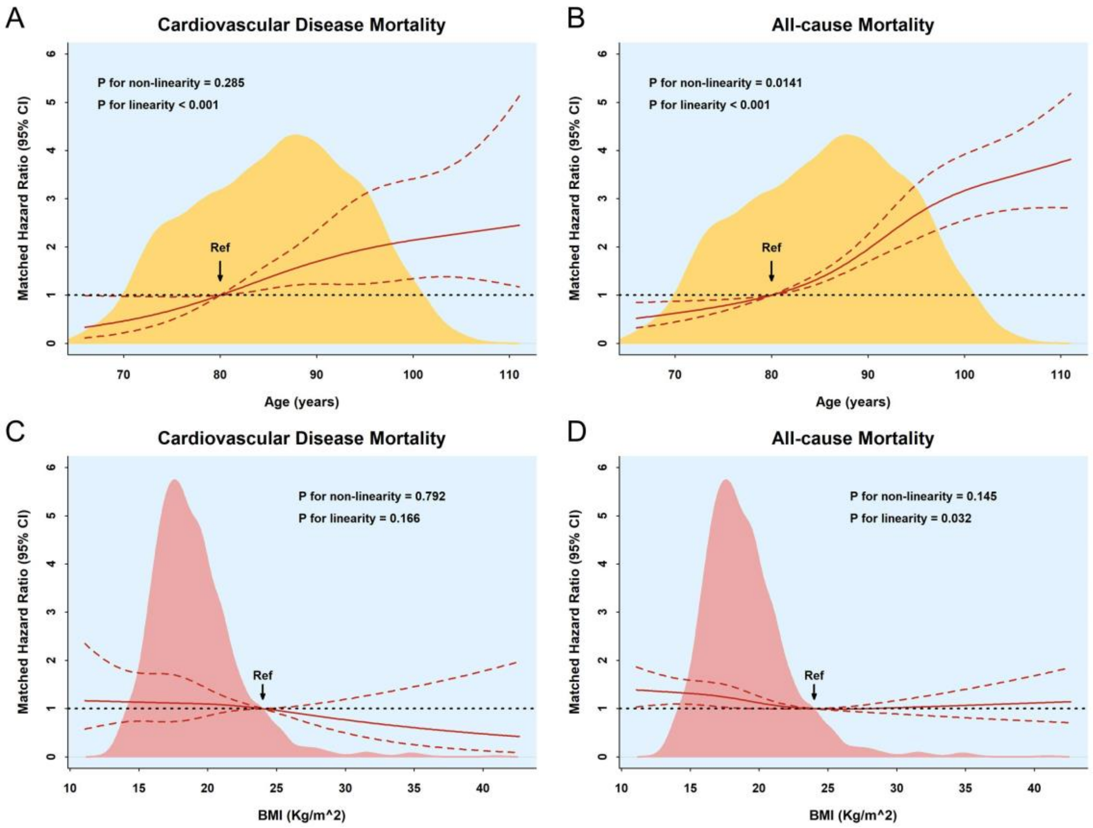

Figure S5 Association between age (A, B) or BMI (C, D) and cardiovascular disease (A, C) and all-cause (B, D) mortality according to RSC regression, with age = 80 years and BMI = 24 Kg/m^2 as reference. The hazard ratio and 95% CI were calculated by adjusting for all covariates. The solid red line indicates the RCS curve and the dashed red line indicates the 95% CI.

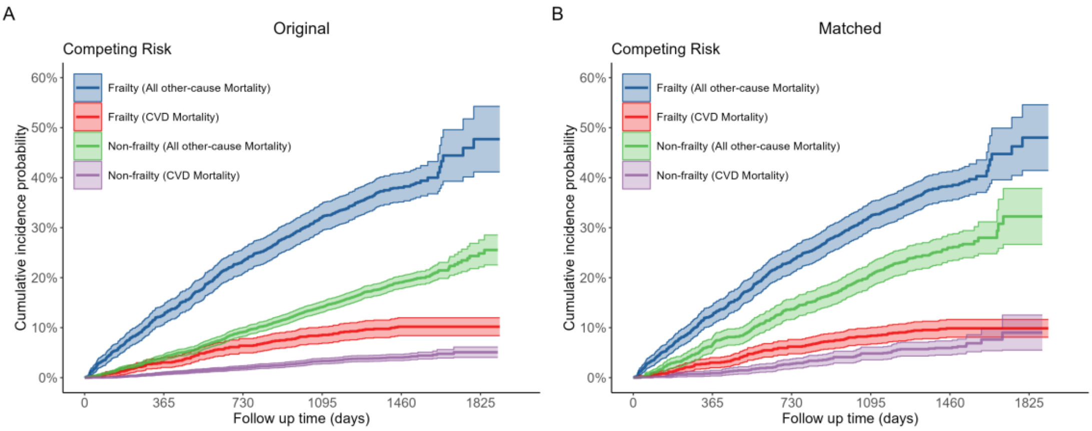

Figure S6 The cumulative incidence probability curves for cardiovascular disease mortality using competing risk models. All other cause mortality events were used as competing risk events.

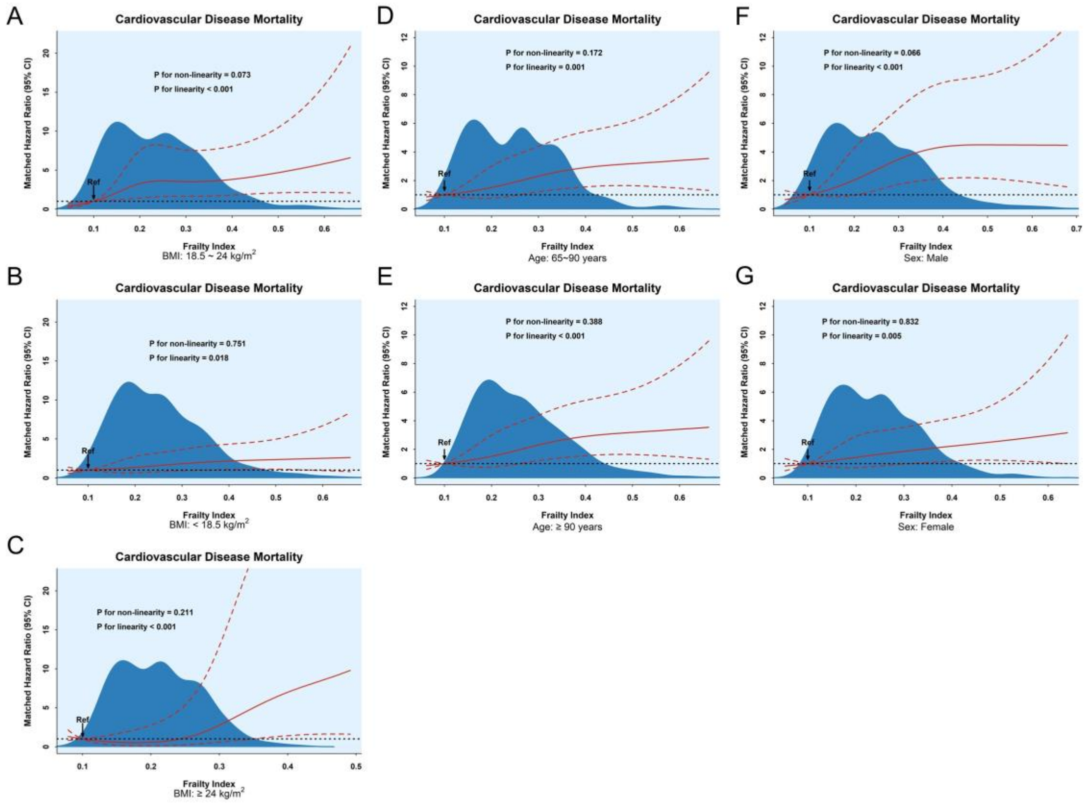

Figure S7 Association between the frailty index and cardiovascular disease mortality according to RSC regression in BMI subgroups (A, B, C), age subgroups (D, E), and sex subgroups (F, G), with frailty index = 0.1 as reference. The hazard ratio and 95% CI were calculated by adjusting for all covariates. The solid red line indicates the RCS curve and the dashed red line indicates the 95% CI.

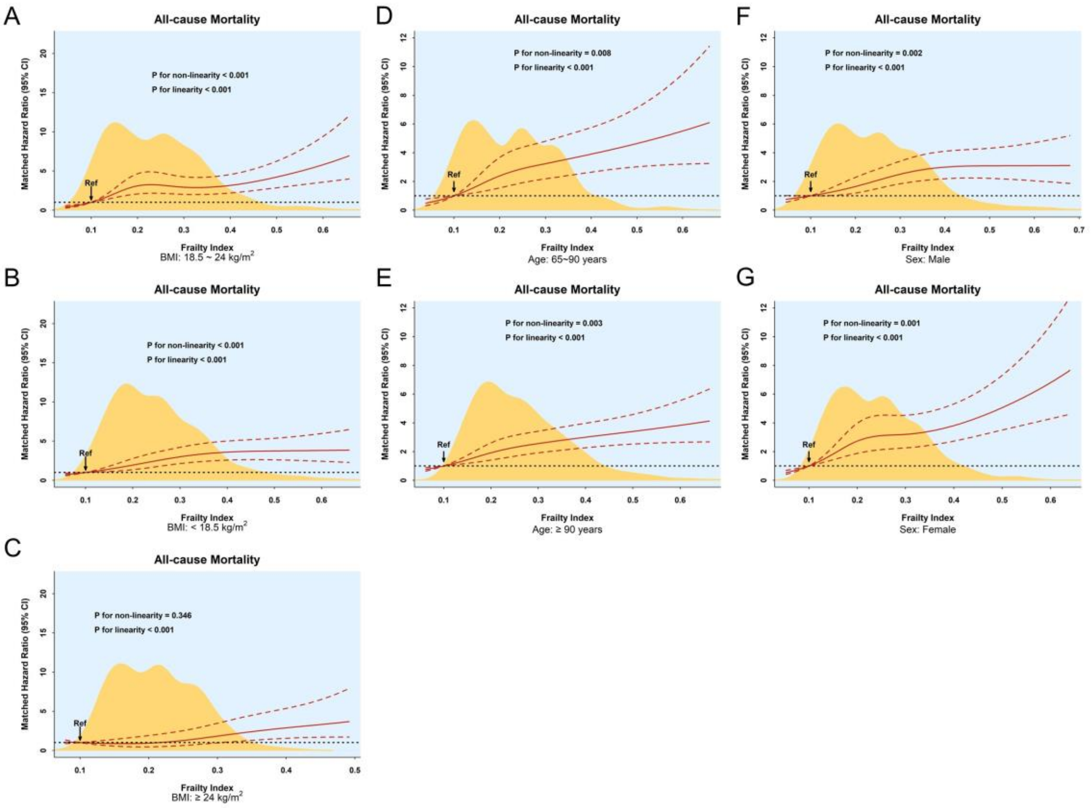

Figure S8 Association between the frailty index and all-cause disease mortality according to RSC regression in BMI subgroups (A, B, C), age subgroups (D, E), and sex subgroups (F, G), with frailty index = 0.1 as reference. The hazard ratio and 95% CI were calculated by adjusting for all covariates. The solid red line indicates the RCS curve and the dashed red line indicates the 95% CI.
